# Identification of druggable genes for multiple myeloma based on genomic information

**DOI:** 10.5808/gi.23011

**Published:** 2023-09-27

**Authors:** Rahmat Dani Satria, Lalu Muhammad Irham, Wirawan Adikusuma, Anisa Nova Puspitaningrum, Arief Rahman Afief, Riat El Khair, Abdi Wira Septama

**Affiliations:** 1 Department of Clinical Pathology and Laboratory Medicine, Faculty of Medicine, Public Health and Nursing, Universitas Gadjah Mada, Yogyakarta 55281, Indonesia; 2 Clinical Laboratory Installation, Dr. Sardjito Central General Hospital, Yogyakarta 55281, Indonesia; 3 Faculty of Pharmacy, Universitas Ahmad Dahlan, Yogyakarta 55164, Indonesia; 4 Research Centre for Pharmaceutical Ingredients and Traditional Medicine, National Research and Innovation Agency (BRIN), South Tangerang 15314, Indonesia; 5 Department of Pharmacy, University of Muhammadiyah Mataram, Mataram 83127, Indonesia; 6 Research Center for Vaccine and Drugs, National Research and Innovation Agency (BRIN), South Tangerang 15314, Indonesia

**Keywords:** biological risk genes, drug repositioning, genomic variants, multiple myeloma

## Abstract

Multiple myeloma (MM) is a hematological malignancy. It is widely believed that genetic factors play a significant role in the development of MM, as investigated in numerous studies. However, the application of genomic information for clinical purposes, including diagnostic and prognostic biomarkers, remains largely confined to research. In this study, we utilized genetic information from the Genomic-Driven Clinical Implementation for Multiple Myeloma database, which is dedicated to clinical trial studies on MM. This genetic information was sourced from the genome-wide association studies catalog database. We prioritized genes with the potential to cause MM based on established annotations, as well as biological risk genes for MM, as potential drug target candidates. The DrugBank database was employed to identify drug candidates targeting these genes. Our research led to the discovery of 14 MM biological risk genes and the identification of 10 drugs that target three of these genes. Notably, only one of these 10 drugs, panobinostat, has been approved for use in MM. The two most promising genes, calcium signal-modulating cyclophilin ligand (*CAMLG*) and histone deacetylase 2 (*HDAC2*), were targeted by four drugs (cyclosporine, belinostat, vorinostat, and romidepsin), all of which have clinical evidence supporting their use in the treatment of MM. Interestingly, five of the 10 drugs have been approved for other indications than MM, but they may also be effective in treating MM. Therefore, this study aimed to clarify the genomic variants involved in the pathogenesis of MM and highlight the potential benefits of these genomic variants in drug discovery.

## Introduction

Multiple myeloma (MM) is a hematological malignancy characterized by the uncontrolled proliferation of abnormal plasma cells in the bone marrow (BM). This abnormal growth of plasma cells inflicts damage on multiple organs throughout the body, resulting in systemic manifestations. These manifestations include hypercalcemia, renal failure, anemia, and bone lytic lesions [1,2]. The number of MM cases has been reported to be on the rise. In 2020, the reported incidence of MM was 160,000 cases, with 106,000 resulting in death [3]. This high mortality rate suggests that the majority of MM cases are fatal. Therefore, to prevent a poor prognosis, it is crucial to have an effective diagnostic tool that can detect the disease at an early stage. Currently, the diagnosis of MM involves a BM analysis to determine the percentage of plasma cells in the BM. This is followed by serum protein electrophoresis for M-band and urinary Bence-Jones protein detection. Subsequently, β-2 microglobulin and serum albumin are used to determine the stage of MM [4,5]. However, these diagnostic tools have proven insufficient for detecting the early stages of MM, with most cases only being identified in the late stages.

More accurate diagnostic tools have recently been developed for the diagnosis of MM and the prediction of its prognosis. One such tool is karyotyping identification, which is utilized to determine the prognosis and treatment plan for this disease [6-8]. However, the application of karyotyping has its limitations, as it can only detect abnormalities at the chromosomal level, not at the gene level. Genomic detection, on the other hand, holds promise for identifying early disease development before it worsens, and it is employed to determine the effectiveness of therapy. Furthermore, it can even be utilized for drug repurposing.

The genome-wide association study (GWAS) Catalog is a database containing the genomic variants associated with various diseases, including MM. While GWAS data have provided valuable biological insights into the genomic variants associated with many diseases, the translation of these insights into clinical situations has remained limited. Therefore, our study aimed to integrate the genomic variants from the GWAS catalog with a bioinformatics-based approach to derive more practical biological insights for MM treatment.

## Methods

### Study design

**Fig. 1.**
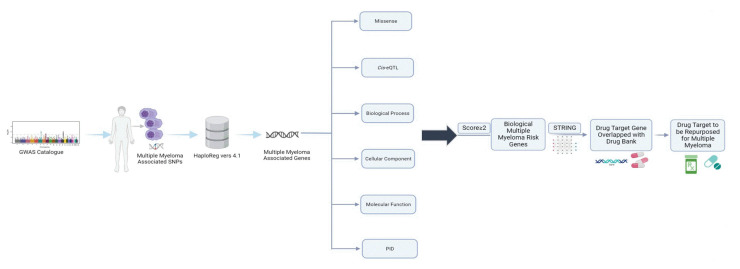
Process of identification for multiple myeloma single nucleotide polymorphisms (SNPs) and the encoded genes driven drug repositioning for multiple myeloma. GWAS, genome-wide association study; PID, primary immunodeficiency.

We began by identifying the genomic variants or single-nucleotide polymorphisms (SNPs) associated with MM using data from the GWAS catalog, with the criterion of a p-value < 10^-8^. Subsequently, we obtained additional SNPs known to encode these genes by leveraging HaploReg version 4.1, focusing on Asian population data from the 1000 Genome Project Phase I. To identify potential MM risk genes, we employed a genomic-driven drug repurposing approach based on established criteria. These genes have been suggested as potential targets for MM treatment. Lastly, we identified prospective drugs where the mechanisms and therapeutic targets intersected. A detailed workflow of the study can be found in Fig. 1.

### MM risk genes

After widening the search parameters with HaploReg version 4.1, we further scrutinized SNPs that encoded genes to identify the biological MM risk genes more precisely. To pinpoint genes with a higher probability and more robust supporting data, we meticulously annotated the biological risk genes. In this study, we ranked the biological MM-risk genes using six distinct criteria. Each gene that met a criterion was awarded 1 point, with a maximum of 6 points per gene. Genes with higher scores were considered to have a greater potential as biological risk genes. We employed six criteria to filter the biological MM risk genes. The first five were as follows: (1) missense mutation, where HaploReg version 4.1 annotated missense mutations in genes containing MM risk SNPs with linkage disequilibrium (r^2^ > 0.80); (2) cis expression quantitative trait loci (cis-eQTL), where MM risk SNP-containing genes exhibited significant cis-eQTL effects in whole blood; (3) biological processes; (4) cellular components; and (5) molecular functions. Criteria 3, 4, and 5 relate to Gene Ontology (GO) categories. We prioritized genes using the Database for Annotation, Visualization, and Integrated Discovery (DAVID) online tool version 6.8 (https://david-d.ncifcrf.gov/tools.jsp) [9]. The sixth criterion was primary immunodeficiency (PID), which was the final annotation used to prioritize the MM risk genes. The International Union of Immunological Societies (IUIS) has compiled PID genes until 2013 [10]. A hypergeometric test was used to analyze the data for enrichment, with a p-value of 0.05 considered significant.

### Discovering new candidate drugs for MM

We utilized a scoring system derived from six criteria to prioritize potential biological MM risk genes. Any genes with scores of 2 or higher were considered candidates. Regrettably, there are only a few druggable target genes. To address this, we expanded our search for biological MM risk genes using the STRING database (https://string-db.org/), accessed on September 12, 2022. After expanding our gene pool based on protein-protein interaction information from the STRING database, we performed an overlap analysis using the DrugBank database, also accessed on September 12, 2022. To validate our findings, we used ClinicalTrials.gov (https://clinicaltrials.gov/; accessed on September 13, 2022) to verify whether the drug target genes were currently under clinical trials. Additionally, we conducted PubMed mining (https://pubmed.ncbi.nlm.nih.gov/; accessed on September 13, 2022) to ascertain whether the candidate drugs were under preclinical investigation.

### Statistical analysis

Analytic workflows were executed using RStudio version 4.2.1 (RStudio, Boston, MA, USA). The haploR package was utilized to identify missense variants and cis-eQTL (https://cran.r-project.org/web/packages/haploR/index.html). GO enrichment analyses, encompassing biological processes, cellular components, and molecular function, were conducted using the RDAVIDWebService. This service is accessible as an R package from the Bioconductor project (www.bioconductor.org) [11].

## Results

### Identification of multiple myeloma-associated genes

In this study, we identified 72 SNPs from the GWAS catalog that met the inclusion criteria of p < 10^-8^ (Supplementary Table 1). We then utilized HaploReg version 4.1, applying a criterion of r^2^ > 0.8 within the Asian population, to expand the SNPs encoding the identified genes. The genomic variants associated with MM were subsequently used to derive the variants encoding these genes. This process led to the identification of 2,555 SNPs that overlapped with 63 genes associated with MM. These genes were then used for further analysis.

### Identification of MM biological risk genes with functional annotation criteria

**Fig. 2.**
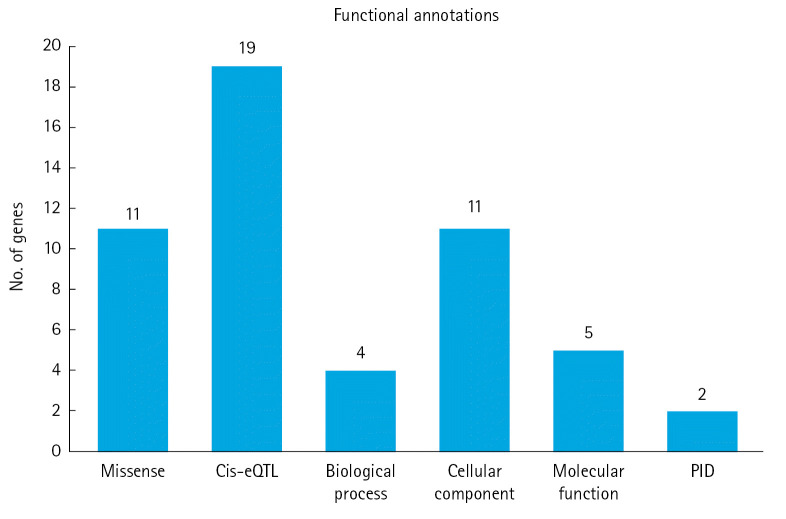
Six functional annotations to prioritize the biological risk genes for multiple myeloma. cis-eQTL, cis expression quantitative trait loci; PID, primary immunodeficiency.

**Fig. 3.**
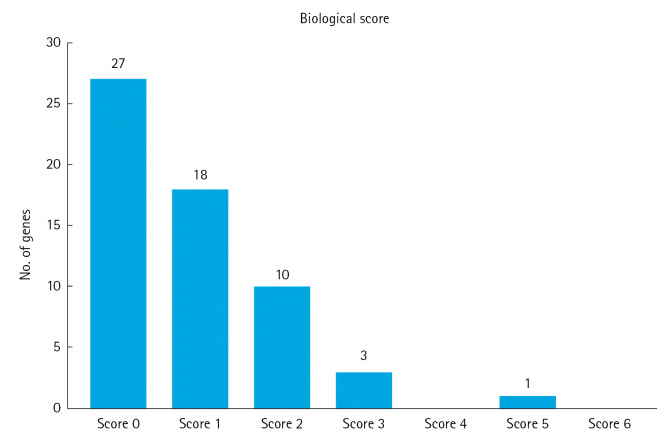
Scoring system for each functional annotation applied. The genes with a score of ≥2 were categorized as “biological multiple myeloma risk genes”.

We utilized six functional annotation criteria to identify genes potentially implicated in the pathogenesis of MM. Each gene was scored based on whether it met each criterion. The criteria included genes with missense variants (n=11), genes with a cis-eQTL effect (n=19), genes categorized as involving a biological process (n=4), genes categorized as involving a cellular component (n=11), genes categorized as involving a molecular function (n=5), and genes categorized as related to PID (n=2) (Fig. 2). Detailed information about the scoring system for each functional annotation is illustrated in Fig. 3. Out of 63 genes, we found that 14 had a score of 2 or more and were thus classified as MM biological risk genes. The top four genes, *RFWD3*, *HMGXB4*, *CDCA7L*, and *CCHCR1*, were identified as the most significant biological risk genes due to their score of 3 or more out of 6 (Table 1). We further expanded our analysis of the 14 MM biological risk genes using the STRING database to identify additional drug-targeted genes. This process yielded 336 gene pairs from the protein-protein interaction network in the STRING database (Supplementary Table 2).

### Candidates for drug repurposing to treat multiple myeloma

**Fig. 4.**
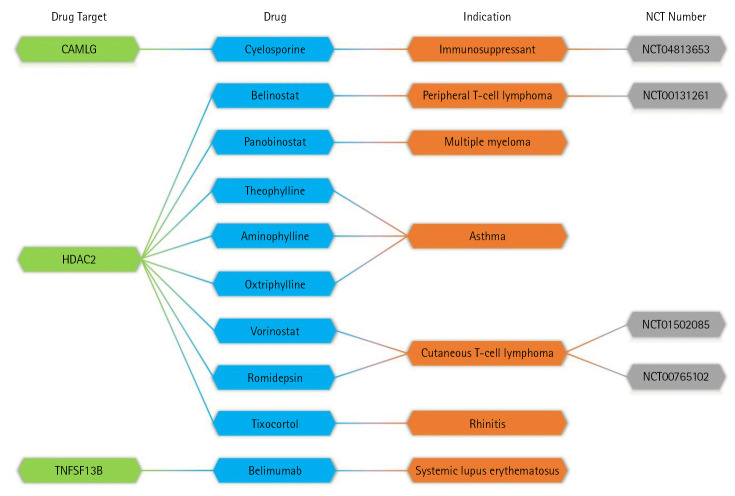
Connections between drug targets and drug candidates for multiple myeloma. *CAMLG*, calcium signal-modulating cyclophilin ligand; *HDAC2*, histone deacetylase 2.

To identify genes targeted by potential drug candidates, we utilized the DrugBank database. It is important to note that not all drugs that target these genes exhibit pharmacological activity. We identified 10 drugs targeting three genes associated with an increased risk for MM. These drugs have already received approval for use in treating other diseases (Fig. 4). Among these 10 drugs, only panobinostat is recognized as an approved drug for MM. Meanwhile, four drugs are currently undergoing clinical trials for MM, and five drugs have not yet been investigated as treatments for MM.

This study focused on drugs that have received approval based on clinical trials, as documented in the ClinicalTrial.gov database. Consequently, the target genes of four drugs currently under clinical investigation—cyclosporine (NCT04813653), belinostat (NCT00131261), vorinostat (NCT01502085), and romidepsin (NCT00765102)—were deemed the most promising for MM treatment. We identified two such target genes: calcium signal-modulating cyclophilin ligand (*CAMLG*) and histone deacetylase 2 (*HDAC2*). Of the five new candidate drugs, four—namely, theophylline, aminophylline, oxtriphylline, and tixocortol—target these promising genes and may also be applicable for MM treatment. The results of this study underscore that human genomic variants not only influence disease risk loci, but can also provide new biological insights for drug repurposing in MM treatment.

## Discussion

In this study, we extracted 72 SNPs associated with MM from the GWAS catalog database, using an inclusion criterion of p < 10^-8^ to search for candidate genes with the potential for drug reuse for MM treatment. We utilized six functional annotations to evaluate and prioritize MM risk genes that could be associated with new drug targets. Our findings revealed three genes targeted by 10 drugs. Of these 10 drugs, panobinostat is the only one currently approved for MM treatment. Meanwhile, four drugs are under clinical investigation for MM, and five drugs have not yet been reported for MM treatment. Two genes, *CAMLG* and *HDAC2*, are targeted by four drugs: cyclosporine (NCT04813653), belinostat (NCT00131261), vorinostat (NCT01502085), and romidepsin (NCT00765102), all of which are currently under clinical investigation. At present, *CAMLG* and *HDAC2* are considered the most promising target genes for MM treatment, as determined by studies and approvals based on clinical trials from the ClinicalTrial.gov database.

Cyclosporine has been shown to be an immunosuppressive agent used in the treatment of postoperative organ rejection [12]. A study by Sonneveld et al. in 1992 [13] demonstrated the clinical utility of cyclosporin in modulating multi-drug resistance in patients with MM, specifically to vincristine, doxorubicin, and dexamethasone. Several target genes have been identified, with belinostat, vorinostat, and romidepsin shown to be effective antineoplastic agents [14-16]. Both belinostat and vorinostat are HDAC inhibitors from the hydroxamate group. Their mechanism of action includes inhibiting growth, influencing cell differentiation, and inducing apoptosis in malignant cells [15].

A clinical study conducted by Plumb et al. in 2003 [17] demonstrated that belinostat exhibits antitumor activity against tumor cells in both *in vitro* and *in vivo* studies. Vorinostat is utilized in the U.S. Food and Drug Administration (FDA)-approved treatment of cutaneous T-cell lymphoma (CTCL) [15]. Furthermore, various studies have indicated that vorinostat can inhibit the growth of tumors, as well as breast and lung cancers [18-20]. Romidepsin is another newly FDA-approved drug for the treatment of CTCL [21]. This was evidenced in phase II studies involving patients with recurrent or refractory CTCL, which showed an overall response rate of 34%–35% [22].

Drug repurposing offers the benefit of exploiting gene variations, utilizing the GWAS catalog database to identify potential new drug candidates for MM [23]. However, this research is not without limitations. In this study, not all the identified target genes exhibited pharmacological activity. Consequently, the identified genes may potentially overlook drug targets previously discovered for MM. Therefore, additional research is necessary to confirm the effects of these candidate drugs in clinical applications for MM disease.

By utilizing the GWAS catalog database to map the relationships between diseases, genes, proteins, and drugs, we identified three drug target genes that could potentially serve as candidates for new MM treatments. We discovered 10 potential drug candidates for MM, and notably, only one approved drug for MM, panobinostat, was identified. Among the targets identified, four drugs are currently undergoing clinical trials for MM, while five drugs have not been reported as MM treatments. Our study revealed that the two most significant biological risk genes for MM are *CAMLG* and *HDAC2*. The evidence suggests a significant association between these genes and MM, warranting further translational research. Drug repurposing presents numerous advantages in the drug development process, including reduced time and costs, and increased success rates. In this study, we merged a drug repurposing approach with an integrative research methodology to identify drugs with new potential applications for MM.

## Figures and Tables

**Table 1.** Functional annotations applied to prioritize the biological risk genes for multiple myeloma

GENCODE_id	GENCODE_name	Missense	Cis-eQTL	Biological process	Cellular component	Molecular function	PID	Total score
ENSG00000168411	*RFWD3*	1	1	1	1	1	0	5
ENSG00000100281	*HMGXB4*	1	0	0	1	1	0	3
ENSG00000164649	*CDCA7L*	0	1	1	1	0	0	3
ENSG00000204536	*CCHCR1*	0	1	1	1	0	0	3
ENSG00000025770	*NCAPH2*	0	0	1	1	0	0	2
ENSG00000080603	*SRCAP*	0	0	0	1	1	0	2
ENSG00000100307	*CBX7*	0	1	0	1	0	0	2
ENSG00000138101	*DTNB*	0	0	0	1	1	0	2
ENSG00000156858	*PRR14*	1	1	0	0	0	0	2
ENSG00000168038	*ULK4*	1	1	0	0	0	0	2
ENSG00000182606	*TRAK1*	0	1	0	1	0	0	2
ENSG00000204525	*HLA-C*	1	1	0	0	0	0	2
ENSG00000204531	*POU5F1*	0	0	0	1	1	0	2
ENSG00000240505	*TNFRSF13B*	1	0	0	0	0	1	2

We established a threshold score of ≥2 from a range of functional annotations numbered from 0 to 6. Each gene was assigned one point for each annotation. Genes with a single functional annotation received one point (score), and those with a score of ≥2 were categorized as "biological multiple myeloma risk genes". Our research indicated that as the threshold of the biological score increased, the quantity of identified biological genes decreased, thereby reducing the number of observable drug targets. For instance, we identified 1 biological multiple myeloma gene for a threshold score of ≥5, 3 biological multiple myeloma genes for a threshold score of ≥3, and 10 biological multiple myeloma genes for a threshold score of ≥2. The more biological multiple myeloma genes we discover, the more potential drug targets for multiple myeloma drug repurposing we can identify. PID, primary immunodeficiency; cis-eQTL, cis expression quantitative trait loci.

## Supplementary Materials

Supplementary Table 1. Seventy-two SNPs associated with multiple myeloma obtained from the GWAS catalog

Supplementary Table 2. Gene pairs from STRING database
