# Supplementary material for: Identification of druggable genes for multiple myeloma based on genomic information
Source: Genomics Inform. 2023 Sep 27;21(3):e31. doi: 10.5808/gi.23011 (PMC10584652; doi:10.5808/gi.23011)
Supplement: Supplementary Table 1. — Seventy-two SNPs associated with multiple myeloma obtained from the GWAS catalog [file gi-23011-Supplementary-Table-1.pdf]

Supplementary Table 1. Seventy-two SNPs associated with multiple myeloma obtained from the GWAS catalog

SNPs	p-value
rs57104699	4,00E-08
rs57104699	2,00E-08
rs6919908	6,00E-10
rs6919908	4,00E-10
rs73071352	3,00E-08
rs57968458	3,00E-10
rs57968458	6,00E-11
rs1050976	6,00E-08
rs3132535	3,00E-17
rs10936600	6,00E-15
rs1052501	4,00E-09
rs34562254	4,00E-17
rs34562254	2,00E-08
rs6595443	1,00E-08
rs4325816	7,00E-09
rs17507636	9,00E-09
rs2790457	2,00E-08
rs58618031	3,00E-08
rs7193541	5,00E-12
rs1948915	4,00E-11
rs11086029	7,00E-11
rs13338946	1,00E-13
rs2811710	2,00E-13
rs877529	1,00E-09
rs56219066	4,00E-08
rs10936599	3,00E-08
rs2285803	1,00E-11
rs1423269	2,00E-11
rs9372120	9,00E-15
rs138740	6,00E-08
rs6746082	2,00E-10
rs7781265	1,00E-08
rs138747	3,00E-08
rs7781265	3,00E-10
rs139402	5,00E-26
rs7577599	1,00E-16
rs56219066	2,00E-10
rs56219066	1,00E-09
rs6599192	9,00E-18
rs4487645	5,00E-15
rs6066835	1,00E-13
rs4273077	3,00E-14

rs1052501	2,00E-08
rs200203825	8,00E-12
rs139371	2,00E-09
rs34229995	1,00E-08
rs4487645	3,00E-14
rs200203825	3,00E-10
rs4487645	1,00E-09
rs2272007	2,00E-09
rs6599175	1,00E-09
rs603965	8,00E-11
rs72773978	7,00E-09
rs603965	2,00E-11
rs2285803	1,00E-10
rs877529	8,00E-16
rs4273077	8,00E-09
rs10936599	9,00E-14
rs12711846	3,00E-14
rs4525246	3,00E-14
rs210143	7,00E-12
rs6763508	8,00E-12
rs12638862	2,00E-11
rs6546149	6,00E-10
rs11715604	2,00E-09
rs9392017	6,00E-09
rs9880772	7,00E-09
rs1875968	9,00E-09
rs51471313	4,00E-08
rs4916473	5,00E-08
rs2720680	7,00E-08
rs131821	7,00E-08

SNP, single-nucleotide polymorphism; GWAS, genome-wide association study.
